# Pollinator shift ensures reproductive success in a camouflaged alpine plant

**DOI:** 10.1093/aob/mcae075

**Published:** 2024-05-09

**Authors:** Tao Huang, Bo Song, Zhe Chen, Hang Sun, Yang Niu

**Affiliations:** State Key Laboratory of Plant Diversity and Specialty Crops, Kunming Institute of Botany, Chinese Academy of Sciences, Kunming, Yunnan 650201, People’s Republic of China; University of Chinese Academy of Sciences, Beijing 10049, People’s Republic of China; State Key Laboratory of Plant Diversity and Specialty Crops, Kunming Institute of Botany, Chinese Academy of Sciences, Kunming, Yunnan 650201, People’s Republic of China; State Key Laboratory of Plant Diversity and Specialty Crops, Kunming Institute of Botany, Chinese Academy of Sciences, Kunming, Yunnan 650201, People’s Republic of China; State Key Laboratory of Plant Diversity and Specialty Crops, Kunming Institute of Botany, Chinese Academy of Sciences, Kunming, Yunnan 650201, People’s Republic of China; State Key Laboratory of Plant Diversity and Specialty Crops, Kunming Institute of Botany, Chinese Academy of Sciences, Kunming, Yunnan 650201, People’s Republic of China

**Keywords:** *Fritillaria delavayi*, alpine ecology, camouflage, plant colour, pollination, pollinator shifting, reproductive success

## Abstract

**Background and Aims:**

There are intrinsic conflicts between signalling to mutualists and concealing (camouflaging) from antagonists. Like animals, plants also use camouflage as a defence against herbivores. However, this can potentially reduce their attractiveness to pollinators.

**Methods:**

Using *Fritillaria delavayi*, an alpine camouflaged plant with inter-population floral colour divergence, we tested the influence of floral trait differences on reproduction. We conducted pollination experiments, measured floral morphological characteristics, estimated floral colours perceived by pollinators, analysed floral scent and investigated reproductive success in five populations.

**Key Results:**

We found that the reproduction of *F. delavayi* depends on pollinators. Under natural conditions, a flower-camouflaged population had 100 % fruit set and similar seed set to three out of four yellow-flowered populations. Bumblebees are important pollinators in the visually conspicuous yellow-flowered populations, whereas flies are the only pollinator in the flower-camouflaged population, visiting flowers more frequently than bumblebees. The camouflaged flowers cannot be discriminated from the rock background as perceived by pollinators, but may be located by flies through olfactory cues.

**Conclusions:**

Collectively, our results demonstrate that the flower-camouflaged population has different reproductive traits from the visually conspicuous yellow-flowered populations. A pollinator shift from bumblebees to flies, combined with high visitation frequency, compensates for the attractiveness disadvantage in camouflaged plants.

## INTRODUCTION

The traits of an organism can be shaped by both mutualists and antagonists (Terhorst *et al.*, 2018). Animals often need to send signals to conspecifics, facilitating mate choice and social interactions, while also needing to conceal themselves (camouflage) to avoid detection by predators or prey. These two requirements inherently conflict with each other (Stuart-Fox and Moussalli, 2009). For instance, the presence of the orange spot on male guppies (*Poecilia rticulata*) is preferred by females but selected against under predation (Andersson, 1994).

A similar scenario also occurs in plants. Unlike animals, most plants rely on vectors for pollination (Tong *et al.*, 2023). Conspicuous floral colours are generally favoured by pollinators but may come at the cost of exposure to antagonists, such as herbivores (Irwin *et al.*, 2003), and nectar or pollen thieves. Consistent with this consideration, when the need for visual communication is absent, plants tend to produce inconspicuous flowers. As an extreme example, wind-pollinated flowers are often brown or green in colour and often lack a perianth (Culley *et al.*, 2002; Friedman and Barrett, 2009). Flowers pollinated by moths (Johnson, 1995), wasps (Shuttleworth and Johnson, 2010), flies (Heiduk *et al.*, 2023) and even rodents (Johnson *et al.*, 2001), which mainly rely on scent cues, are often cryptic in colour. Alternatively, some South African Asteraceae species use bright flower colours to attract pollinators during the daytime, while closing the flowers when pollinators are inactive to display a cryptic lower petal surface, decreasing conspicuousness to herbivores (Kemp and Ellis, 2019). Utilizing the different colour vision capacity of mutualists and antagonists, red-coloured bird-pollinated flowers can attract birds and decrease detection by bees (less efficient visitors lacking the red photoreceptor) simultaneously (Raven, 1972; Chen *et al.*, 2020). In addition, the flower aposematism hypothesis suggests the vivid colours and certain scents of poisonous flowers not only attract pollinators but also function as a warning signal to deter potential herbivores (Hinton, 1973; Lev-Yadun, 2024).

Plants usually utilize mechanical (Lucas *et al.*, 2000) and chemical weapons (Mithöfer and Boland, 2012) in response to herbivores, while camouflage has also been reported as a defensive strategy in plants over the last decade (Burns, 2010; Lev-Yadun, 2016; Niu *et al.*, 2018). In New Zealand, *Pseudopanax crassifolius* (Araliaceae) exhibits an ontogenetic leaf colour change from cryptic seedling leaves to green adult leaves, which was suggested as an evolutionary response to giant browsing birds (Fadzly *et al.*, 2009). In California, the colour of *Streptanthus breweri* (Brassicaceae) resembles the local soil outcrops, and a colour mismatch between the leaf and substrate increased damage rates (Strauss and Cacho, 2013). In Southwest China, the rock-like leaves of *Corydalis benecincta* (Papaveraceae) result in higher survivorship under the selection pressure of herbivores (Niu *et al.*, 2014). The seeds of *Pinus halepensis* (Pinaceae) exhibit bimodal coloration, considered as a cryptically adaptive strategy under predation pressure by seed consumers (Lev-Yadun and Ne’eman, 2013). Most known camouflaged plants rely on animals for pollination. Despite having camouflaged leaves, they need to produce bright flowers in certain seasons to attract pollinators, exposing themselves to potential herbivores. This essentially reflects a conflict between survival and reproduction. To the best of our knowledge, the only study that has touched on this topic focused on *Monotropsis odorata* (Ericaceae), a mycoheterotrophic plant found in North America (Klooster *et al.*, 2009). This plant is covered by cryptic bracts, decreasing its detectability to herbivores, while it was suggested that fragrant floral scent still attracts bumblebees for pollination (Klooster *et al*., 2009). However, the potential influence of cryptic bracts on its visual attractiveness to pollinators was not quantified.

**Fig. 1.**
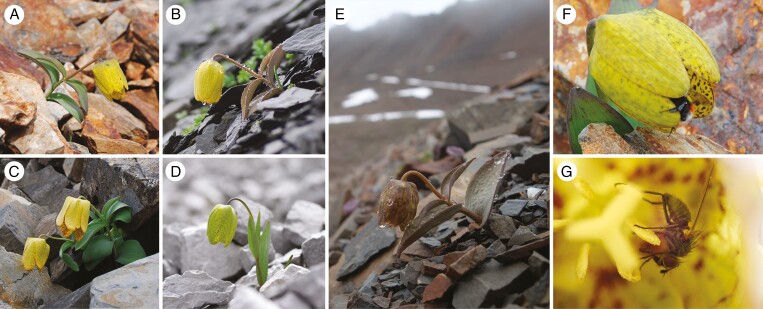
*Fritillaria delavayi* (Liliaceae) with various floral colours and its pollinators in different populations. Non-camouflaged flowers in populations HS, PY, HLH and TBS, respectively (A–D). Camouflaged flower in population PJ (E). Bumblebee (*Bombus prshewalskyi*) visiting a yellow flower (F). A fly (Anthomyiidae) visiting a camouflaged flower (G). Photo credits: (A, G) Zemin Guo, (B–E) Yang Niu, (F) Wanyuan Dang.

In the biodiverse Hengduan Mountains of SW China, camouflage through background matching has been observed in many plants from various taxa (Niu *et al*., 2018; Huang *et al.*, 2023). The driving force behind this camouflage is attributed to selection pressure from herbivores (Niu *et al*., 2014, 2017) or even from commercial plant collection by humans (Niu *et al.*, 2021). *Fritillaria delavayi* (Liliaceae) is one of these camouflaged plants found in alpine screes at about 4000 m a.s.l. The leaf colour of this plant diverges between populations, from green to cryptic (grey or brown), associated with local harvest pressure (Niu *et al*., 2021). Similar locally adapted camouflage has also been found in *Acmispon wrangelianus* (Fabaceae) (Porter, 2013) and *Corydalis hemidicentra* (Papaveraceae) (Niu *et al*., 2017). Different from other camouflaged plants, the floral colour of *F. delavayi* also varies among populations. In most populations, the plant produces yellowish flowers (Fig. 1A–D), whereas in a flower-camouflaged population, it has flowers matching the colour of the rock background (Fig. 1E). A yellow-flowered population was reported to be pollinated by bumblebees (Gao *et al.*, 2014), an important pollinator group in the alpine zone (Bingham and Orthner, 1998) that locates flowers primarily using colour signals (Odell *et al.*, 1999). However, we have no idea how the cryptic flowers are pollinated in the flower-camouflaged population, and specifically whether the cryptic floral colour influences reproductive success.

We propose three predictions. First, given the low attractiveness to pollinators, the flower-camouflaged population has evolved autogamy for reproductive assurance. Second, it exhibits lower reproductive fitness compared to yellow-flowered populations, potentially due to lower attractiveness to pollinators, suggesting a trade-off between survival and reproduction. Third, the flower-camouflaged population relies on a different pollinator group that does not depend on visual cues, ensuring reproductive success. To test these predictions, we conducted pollination experiments, measured floral morphological characteristics, estimated floral colours perceived by different pollinators, analysed floral scent and investigated reproductive successes in five populations.

## MATERIALS AND METHODS

### Plant species and study sites

*Fritillaria delavayi* Franch. (Liliaceae) is a perennial herb found in the alpine screes of the Hengduan Mountains, ranging from 3700 to 5600 m a.s.l (Xu *et al.*, 2014). Typically, it produces a solitary, nodding flower from May to June, with a floral longevity of about 11 d (Gao *et al*., 2014). Nectar drops can be found at the lower part of the tepal. The ovule number for *F. delavayi* ranges from 41 to 257 (with a mean of 91). Our previous work has revealed that leaf colour varies among populations, from normal green to camouflaged (grey and brown) (Niu *et al*., 2021). Flower colours also vary among populations, from yellow to camouflaged. A study based on a single population suggests *F. delavayi* is mainly pollinated by bumblebees (Gao *et al*., 2014). *Fritillaria delavayi* is a traditional medicinal herb and faces the threat of destructive commercial harvesting, particularly targeting its bulbs (Niu *et al*., 2021). Since 2021, it has legal status as a National Class II Protected Plant in China. Our experiments were permitted by the Government of Diqing Tibetan Autonomous Prefecture [Plant Collection Certificate Numbers (Di): 2022145 and 2023196].

We conducted experiments in five populations in NW Yunnan province, SW China, from May to July during 2021–2023. Detailed information for each population is shown in Supplementary Data Table S1. Four populations, namely HS, TBS, PY and HLH, produce yellow flowers, regardless of their leaf colour (Fig. 1A–D). Plants from population PJ produce dark-brown flowers that closely match the surrounding rocks, forming good camouflage (Fig. 1E). Ideally, we required more populations with camouflaged flowers, but based on a comprehensive survey, PJ is the only population we found for observation.

### Estimation of reproductive success

To estimate whether the reproduction of *F. delavayi* depends on pollinators, 20–50 flowers from each population were tagged and netted using mesh bags to exclude pollinators per year. However, only 4–21 samples were eventually obtained over the 3 years because of heavy commercial harvest. As natural controls, 20–60 individuals from each population were tagged and left open to pollinators per year, and 4–58 samples were eventually recaptured. Fruits were harvested at maturity, typically in August. Each fruit underwent dissection to distinguish viable from aborted seeds. The aborted seeds were characterized as those that did not fully expand and were found to be empty.

Fruit set was determined by dividing the number of fruits by the total number of flowers. For plants that produced fruits, we calculated seed production as the total number of viable seeds per plant. Subsequently, seed set was calculated as the number of viable seeds divided by the total number of ovules. Chi-square tests were used to examine the differences in fruit set between populations. One-way ANOVAs were used to analyse the differences in seed production and seed set, followed by Tukey’s tests.

### Pollinator assemblage and visitation frequency

To investigate the variation in pollinator assemblage among populations, we conducted comprehensive field observations of *F. delavayi* floral visitors across the five populations mentioned above. In the flower-camouflaged population PJ, observation was conducted for three successive years, from 2021 to 2023. For the yellow-flowered populations, observations were conducted in all four populations in 2023, but some were missed in 2021 and 2022 because of the Covid-19 pandemic. About 15 flowering individuals were observed per year per population. To compare the flower visitation frequency across populations, we recorded the number of flower visits in 30-min intervals, observing one to five individuals simultaneously within each interval.

Flower visitor observation was conducted from 1000 to 1800 h in each population under good weather conditions. Occasionally, intermittent brief rain showers disrupted observations, but the entire observation period spanned across different times throughout the day. For each visit, we recorded the visitor species (identified later), behaviour at the flower (nectar feeding, pollen feeding or pollen collection), and whether the insect contacted the anthers and stigma. In total, observations were conducted for 28–80 h for each population. Insect visitors landing on flowers were captured using mesh bags and examined by a cryo-scanning electron microscope to confirm whether the pollen attached to insects belonged to *F. delavayi*. All flower visitors were identified at least at the family level. Insects that (1) visit flowers with substantial frequency, (2) contact the anthers and stigmas, and (3) are large enough to carry a significant amount of pollen were considered as pollinators. Visitation frequency was interpreted as the number of pollinator visits per flower per hour. For the year 2023, differences in visit frequency between populations were analysed using one-way ANOVA. For the year 2022, with only two populations observed, an independent samples *t*-test was applied. Data were square-root transformed to enhance normality.

### Morphological measurements of flowers and pollinators

To examine morphological differences between populations, especially between camouflaged and yellow flowers, we measured flower diameters, the length and width of the perianth, as well as the length of stamen and pistil using a digital caliper (with precision of 0.01 mm, 11–24 flowers per population). We first measured flower diameter using intact flowers. Subsequently, we dissected the flowers and laid out the petals, stamens and pistils flat on the experimental table for further measurement of other morphological indicators. Each individual was measured three times before averaging. We also calculated the distance between stamen and pistil as the length of the pistil minus the length of the stamen, as an estimation of self-fertilization capacity across populations. Larger pistil–stamen values are suggestive of a weaker self-pollination capacity. Differences in these parameters between populations were assessed using one-way ANOVA, followed by Tukey’s tests. We applied a log or square-root transformation to improve normality (perianth width: log-transformed; stamen–pistil distance: square-root transformed). As references, we also measured the body length of the main pollinators caught in the corresponding populations, i.e. two *Bombus* species (six *B. prshewalskyi* individuals and three *B. friseanus* individuals) and three Anthomyiidae species (24 individuals) using a digital caliper. We provide precise measurements of flower morphological characteristics and pollinator body sizes in the Supplementary Data.

### Reflectance spectra measurements

Floral colour was measured in terms of the reflectance spectra using 10–15 flowers for each population. Newly opened flowers were collected and preserved in a car refrigerator, then measured within 3 h. For each population, we also collected 10–15 pieces of rocks that were close to the plant as the background of the flowers. Reflectance spectra were measured using a spectrometer (FLAME, Ocean Optics) equipped with a UV-VIS light source (DH2000 BAL, Ocean Optics). The raw spectra were read from 180 to 875 nm, with 0.39-nm resolution. These raw data were then processed into 1-nm intervals from 300 to 700 nm using the R package ‘pavo’ (Maia *et al.*, 2013). The fibre-optics probe was fixed in a black holder (RPH-1, Ocean Optics) at 45° (for both illumination and collection). The distance between each sample and the probe was maintained at ~5 mm. A polytetrafluoroethylene-based optical diffuser (WS-1, Ocean Optics) served as the white standard. To ensure that we estimated the visible part for pollinators, the outer surface of flowers and upper surface of rock substrates were measured. We randomly selected three petals for reflectance spectra measurements. Each sample was measured three times by removing the probe and replacing it after each measurement, and then averaged for subsequent analyses. All reflectance spectra were first processed using the R package ‘pavo’ (Maia *et al.*, 2013) and later analysed in colour vision models.

We utilized non-metric multidimensional scaling to reduce the dimension of spectral data variables (300–700 nm with 1-nm resolution, 400 variables). The colour divergence among populations was tested using PERMANOVA based on Bray–Curtis similarity. Additionally, we used the ‘pairwise.adonis’ function (Factor: population; Permutations = 100 000) in the ‘vegan’ R package (Oksanen *et al.*, 2007) to conduct pairwise comparisons between populations and determine the significance of differences in each pair of populations.

### Flower conspicuousness as perceived by pollinators

Bumblebees and flies are the main pollinators of *F. delavayi*. To quantify the conspicuousness of flowers to bumblebees, we calculated the chromatic contrast between the flowers and background scree using the Colour Hexagon model (CH model) (Chittka, 1992). This is a tri-chromatic vision model, widely adopted for Hymenoptera, that represents colours as points in space based on the photoreceptor excitation they induce (Chittka, 1992). The colour contrast is determined by the Euclidean distance between colour loci in the CH model, with greater colour distance indicating stronger contrast (Chittka, 1992). It has been suggested that two colours with a distance <0.11 hexagon units cannot be distinguished by bees (Dyer and Chittka, 2004). Given that spectral sensitivity is conservative in bees, we used the sensitivity curve of *Bombus terrestris* (Skorupski *et al.*, 2007).

Troje’s categorical fly colour vision model (Troje, 1993) was used to estimate colour perception by Anthomyiidae flies. This model, established for dipteran insects, is based on the behavioural data of the blowfly *Lucilia* sp. (Troje, 1993). It assumes that colours falling within the same quadrant are indistinguishable by flies (Troje, 1993). However, recently an increasing body of research has demonstrated the discernment of small colour differences by flies, identifying the minimum colour distance (0.021 Troje units) discriminable by hoverflies (**Eristalis* tenax*) (Hannah *et al.*, 2019). Consequently, we also calculated the chromatic contrast between flowers and background screes in Troje’s model. As sensitivity curves of Anthomyiidae flies are not available, we used the data of the blowfly *Lucilia* sp. (Calliphoridae) (Lunau, 2014) instead, which shares the closest phylogenetic relationship with Anthomyiidae flies (Zhang *et al.*, 2015; Xing *et al.*, 2023). Details of the modelling protocol can be found in Shrestha *et al*. (2016). Colour distance >0.096 Troje units are easily discriminable to flies (Garcia *et al.*, 2022). Similarly, in the CH model, the colour discrimination threshold of bumblebees (0.11 hexagon units) is also easily distinguishable, allowing for comparable results.

In both models, we employed an average of 10–15 background rocks reflectance spectra specific to each population as the background spectrum. CIE standard illuminant D65 (daylight) was used for the irradiance spectrum, which reflects the field condition of an open sunny environment. Differences in chromatic contrast (colour distance, square root transformed) between flowers and backgrounds among populations were analysed using one-way ANOVA, followed by Tukey’s tests. One-sample *t*-tests were used to test if the flower–rock mean colour distances are higher than the colour discrimination threshold for bumblebees (0.11 hexagon units) or flies (0.096 Troje units).

### Chemical characterization of floral odour

Four to five newly opened flowers were individually collected, and placed in a separate polyethylene-based fresh bag, which was securely sealed with twine and preserved in a car refrigerator. The flower volatiles from each individual were extracted and identified using a gas chromatograph-mass spectrometer (HP6890GC/5973MS; Agilent Technologies, USA) across five populations (four or five individuals per population). During the preparation stage, each flower sample was placed in a headspace vial, and 0.5 μg ethyl caprate was added as an internal standard. After sealing, a solid-phase microextraction column (65 μm PDMS/DVB, Fused Silica 24Ga, Manual Holder, 3pk; SUPELCO, USA) was used to adsorb the flower odour headspace samples at room temperature, a process that lasted 50 min. Negative controls (empty headspace vial: *n* = 2; headspace vial containing leaf: *n* = 1) were gathered to detect environmental chemical contamination using a uniform protocol.

For gas chromatography, an HP-5MS quartz capillary column with helium as the carrier gas (30.0 m × 0.25 mm × 0.25 μm; Agilent) was employed. The column temperature initially was 40 °C, then increased to 80 and 280 °C at a rate of 3 and 5 °C min^−1^, respectively. The final temperature was maintained for 10 min. The injector was set to a split ratio of 2:1. The column flow rate was 1 mL min^−1^, the inlet temperature was 250 °C and the column front pressure was 100 kPa.

For mass spectrometry, the mass spectra were taken at 70 eV (in EI mode), scanning from *m*/*z* 35 to 350. The transmission line, MS source and quadrupole temperatures were set at 250, 230 and 150 °C, respectively. Compound identification was achieved by comparing the mass spectra and retention indices with standard mass spectral libraries (Wiley7n.l; NIST98.L). The relative percentages of each compound were determined using the total ion current signal. Through the internal standard, we calculated the mass of focal floral scent compounds. Before data analysis, all compounds found in blank and green leaf controls were subtracted from each sample.

Differences in flower scent composition among populations were tested using the ‘adonis’ function in the ‘vegan’ R package (Oksanen *et al*., 2007). Additionally, we employed pairwise adonis (Factor: population; Permutations = 100 000) to conduct pairwise comparisons between populations and determine the significance of differences in each pair of populations. Flower volatile composition was visualized using non-metric multidimensional scaling based on Bray–Curtis dissimilarities. To assess the effectiveness of the specific configuration in generating the observed distance matrix, we used the stress value. Typically, a stress value <0.2 is ideal. The difference in the total amount of floral volatiles among populations was analysed through one-way ANOVA, followed by Tukey’s tests. All analyses were performed in R v.4.1.1 (R Core Team, 2009).

## RESULTS

### Pollinator dependency and reproductive success

*Fritillaria delavayi* faced extensive commercial harvesting, making it challenging to relocate the labelled individuals during the fruit season. To include enough samples, we pooled bagged samples from all three years. All bagged flowers (pollinators excluded) failed to set fruit (*n* = 20 in TBS, 21 in HLH, four in HS, eight in PY and 16 in PJ), indicating that pollinators are essential for reproductive success.

**Fig. 2.**
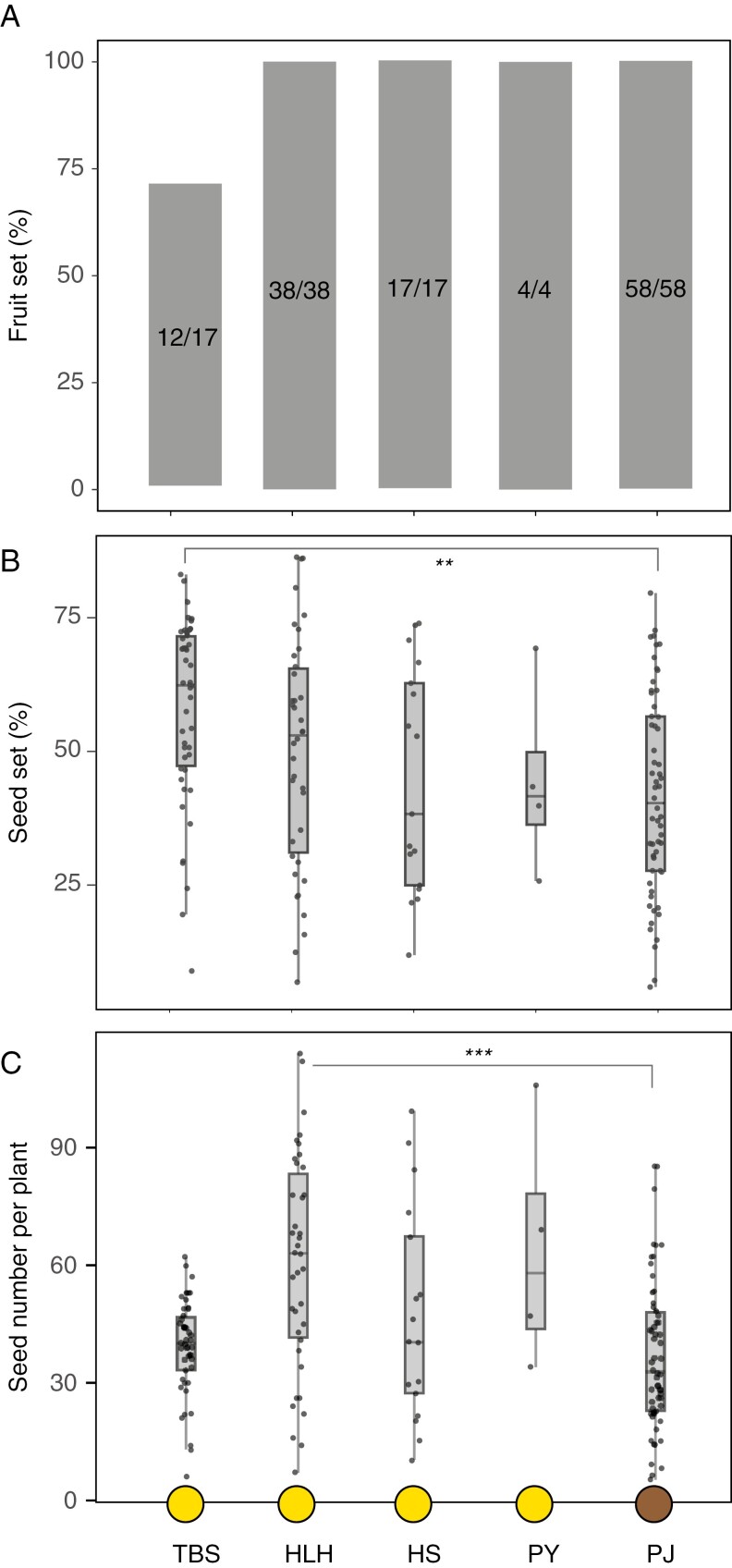
Reproductive success of *F. dedavayi* across populations in terms of fruit set (A, with sample sizes shown within bars), seed set (B) and seed number per plant (C). Yellow circles at the bottom represent non-camouflaged populations, and the brown circle indicates the camouflaged population. Bars indicate standard errors. Asterisks indicate significance differences at the **0.01 and ***0.001 probability level.

Under natural conditions, fruit set was 100 % in four of the five populations we studied, including the flower-camouflaged population PJ (chi-square test, χ^2^ = 35.746, d.f. = 4, *P* < 0.001, Fig. 2A). The yellow-flowered population TBS had lower fruit set than other populations (19/32 in 2022, Supplementary Data, chi-square test, χ^2^ = 18.958, d.f. = 3, *P* < 0.001, Fig. S1; 12/17 in 2023, Fig. 2A).

Seed set in the four populations (TBS, HLH, HS, PJ) studied in 2022 ranged from 47 to 61 %, without no significant difference between them (one-way ANOVA, *F*_3,82_ = 1.38, *P* = 0.255, Supplementary Data, Fig. S1). Seed set for the five populations studied in 2023 ranged from 42 to 57 % (Fig. 2B). One-way ANOVA showed a significant difference between populations (*F*_4,158_* *= 4.295, *P* = 0.002, Fig. 2B), but this was merely due to lower seed set in PJ (flower-camouflaged) than TBS (yellow-flowered) (*P* < 0.001, Tukey test). In other words, seed set in the flower-camouflaged population PJ was similar to three out of the four yellow-flowered populations.

Seed number per plant differed among populations in both 2022 (one-way ANOVA, *F*_3,82_ = 3.654, *P* = 0.02, Supplementary Data, Fig. S1) and 2023 (one-way ANOVA, *F*_4,158_ = 5.191, *P* = 0.001, Fig. 2C), but this was merely due to higher seed production in HLH. In other words, seed number per plant in the flower-camouflaged population PJ was comparable to that of the three yellow-flowered populations in 2022 (Fig. S1) and 2023 (Fig. 2C).

### Pollinators and visit frequency

Both Hymenoptera (Tenthredinidae, Braconidae, *Bombus*, *Hylaeus*) and Diptera (Anthomyiidae, Sciaridae, Phoridae and Bibionidae) insects were observed visiting *F. delavayi*. Braconidae, Bibionidae, Sciaridae, Phoridae and *Hylaeus* visited flowers for nectar, but only at low frequency and did not contact anthers or stigma. Therefore, these insects were classified as nectar robbers rather than pollinators. Tenthredinidae were pollen thieves and visited flowers at very low frequency.

*Bombus* spp. and Anthomyiidae spp. were the most frequent visitors in 2022 (59.7 %) and 2023 (95.7 %). Bumblebees visited flowers for nectar (Fig. 1F), facilitating pollen transfer. Anthomyiidae flies visited flowers for pollen and nectar (Fig. 1G), and sometimes mated inside flowers. They contacted both anthers and stigmas during these processes. Therefore, both bumblebees and Anthomyiidae were considered pollinators of *F. delavayi*, but their composition differed among populations.

**Fig. 3.**
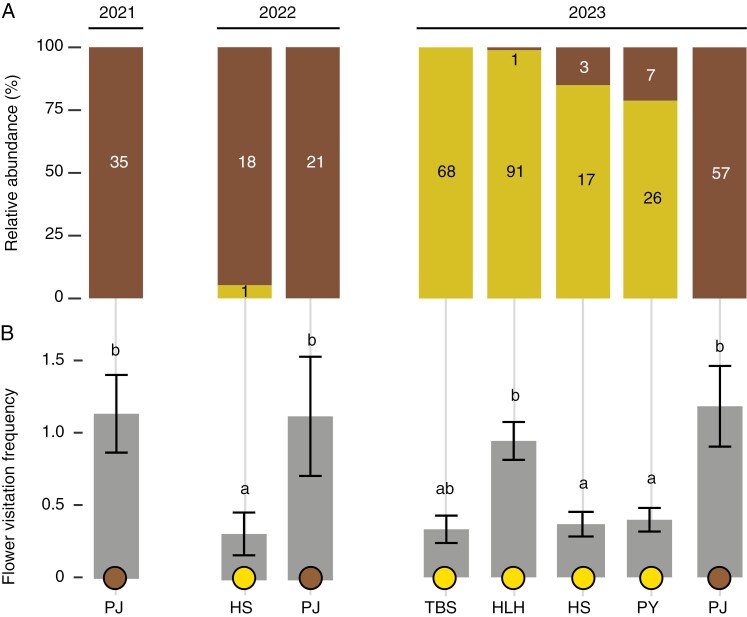
Pollinator composition (A) and their visitation frequencies (B) across populations from 2021 to 2023. Yellow bars represent bumblebees, and brown bars indicate flies. Yellow circles at the bottom represent yellow-flowered populations, and the brown circle indicates the flower-camouflaged population. Numbers within bars are the corresponding visitation counts by pollinators. Different letters above the bars indicate significant differences (*P* ≤ 0.05).

As shown in Fig. 3A, a notable pattern is that bumblebees occurred in the pollinator assemblies of all four yellow-flowered populations, but were absent in the flower-camouflaged population PJ. In 2023, bumblebees were the only pollinator in TBS (with yellow flowers). By contrast, for all three years we observed (2021–2023), flies were the only pollinators in the flower-camouflaged population PJ.

In 2023, visitation frequency was 0.33, 0.94, 0.37 and 0.40 per flower per hour for the four yellow-flowered populations TBS, HLH, HS and PY respectively, and 1.18 for the flower-camouflaged population PJ (Fig. 3B). This frequency differed significantly between populations (one-way ANOVA; *F*_4,436_ = 6.397, *P* < 0.001). Notably, the flower-camouflaged population PJ had significantly higher visit frequency (by Anthomyiidae) than that in two of the four yellow-flowered populations HS and PY, with the exception of TBS and HLH (Fig. 3B). This pattern was consistent with the 2022 findings, where flower visitation frequency in the flower-camouflaged population was significantly higher than in the non-camouflaged HS population in 2022 (Welch’s *t*-test, *t* = −2.33, d.f. = 33.85, *P* = 0.03) (Fig. 3B).

### Floral morphological characteristics

There were significant differences in flower diameter, perianth length, perianth width, pistil length and stamen length of *F. delavayi* flowers between populations (Supplementary Data, Table S2). Specifically, flower diameter of the flower-camouflaged population PJ was significantly smaller than those of yellow-flowered populations (HLH, HS, PY and TBS) (Table S2). However, stamen–pistil distance showed non-significant differences among populations (Table S2), implying that their capacity for self-pollination was similar.

Accordingly, Anthomyiidae pollinators were much smaller than the two bumblebee species (5.83 ± 0.27 mm vs. 28.03 ± 0.79 and 17.07 ± 0.60 mm, Supplementary Data, Fig. S2), which made them more suitable for pollination in the smaller flowered population PJ.

### Flower colour perceived by pollinators

**Fig. 4.**
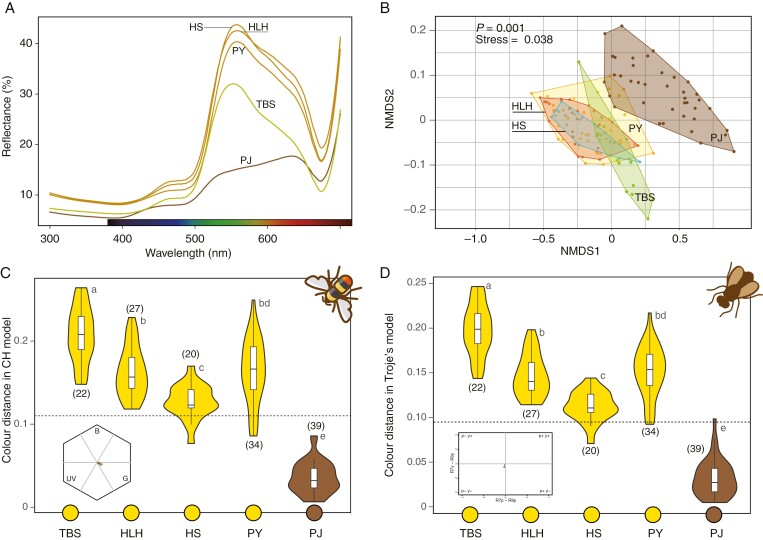
Floral colour properties across populations. Averaged spectral reflectance of flowers (A). Spectral divergence between populations analysed by non-metric multidimensional scaling (B). The colour distance between flowers and rocks calculated using the bee colour hexagon model (C). The dashed line indicates 0.11 hexagon units, the discrimination threshold of bumblebees (Dyer and Chittka, 2004). Colour distance between flowers and rocks (D) calculated in the fly colour model (Troje, 1993; Hannah *et al*., 2019). The dashed line indicates the fly threshold of 0.096 Troje units. Yellow circles at the bottom represent yellow-flowered populations, and brown colours indicate the flower-camouflaged population. Numbers in parentheses are sample sizes. Different letters above the bars indicate significant differences (*P* ≤ 0.01).

There were significant differences in reflectance spectra between the populations we studied (PERMANOVA, *F*_4,137_ = 44.20, *R*^2^ = 0.56, *P* = 0.001) (Fig. 4A, B). The yellow-flowered populations (HLH, HS, PY, TBS) were nested within each other. Among these, the flower-camouflaged population PJ showed the most pronounced divergence from the non-camouflaged populations (pairwise adonis, *P* < 0.001 for all pairs between PJ and other populations, Fig. 4B).

In the bumblebee colour vision model (hexagon model), the colour distances between flowers and background scree varied significantly across populations (one-way ANOVA; *F*_4,137_ = 189.8, *P* < 0.001, Fig. 4C). In the four yellow-flowered populations, the mean chromatic contrast between flowers and rocks significantly exceeded 0.11 hexagon units (one-sample *t*-test, *t* = 13.56, d.f. = 102, *P* < 0.001, Fig. 4C), indicating that bumblebee pollinators could distinguish these flowers from the background scree. By contrast, this averaged chromatic contrast in PJ was only 0.035 CH units, far below the discrimination threshold (one sample *t*-test, *t* = −24.706, d.f. = 38, *P* < 0.001).

In the fly colour vision model, colour distances between flowers and background scree varied significantly across populations (one-way ANOVA; *F*_4,137_ = 169.4, *P* < 0.001, Fig. 4C). In the four yellow-flowered populations, the chromatic contrast between flowers and rocks significantly exceeded 0.096 Troje units (one-sample *t*-test, *t* = 18.355, d.f. = 102, *P* < 0.001, Fig. 4C), indicating that fly pollinators could easily distinguish the flowers from background scree. By contrast, the chromatic contrast between flowers and background screes in PJ (mean colour distance = 0.037 Troje units) was significantly lower than the threshold (one-sample *t*-test, *t* = −17.101, d.f. = 38, *P* < 0.001, Fig. 4C), indicating that fly pollinators could not accurately discriminate the flowers from background scree.

### Flower scent

*Fritillaria delavayi* flowers emitted a delicate, pleasant aroma that was perceptible by us throughout their blooming periods. We detected a total of 32 compounds belonging to four classes (details shown in Supplementary Data, Table S3). Among the identified compounds, terpenoids were the most abundant (17 compounds, 53 %), followed by fatty acid derivatives (seven compounds, 22 %), benzenoids (six compounds, 19 %) and nitrogen-containing compounds (two compounds, 6 %).

Camouflaged flowers in population PJ contained a more diverse range of terpenes (15 compounds) than the yellow-flowered populations (five compounds). The most abundant individual compounds in PJ were *cis*-farnesol, diisobutyl phthalate, d-germacrene, caryophyllene and styrene. These five compounds contributed an average of 61.7 % to the total scent compounds in the PJ population.

**Fig. 5.**
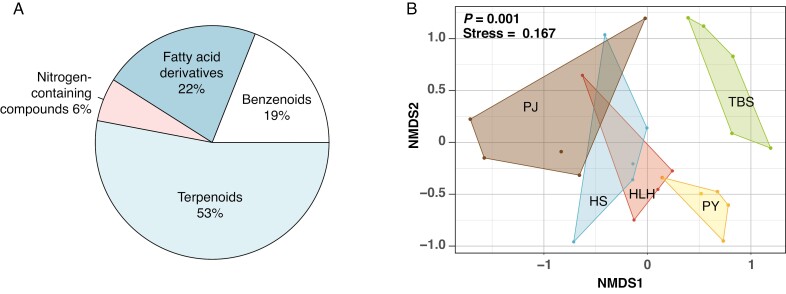
Floral volatile composition (A) and divergence across populations analysed by non-metric multidimensional scaling (B).

The total amount of floral volatiles was similar among populations (one-way ANOVA; *F*_4,19_* *= 2.696, *P* = 0.062). Floral scent composition differed significantly among the populations (PERMANOVA, *F*_4,19_ = 3.49, *R*^2^ = 0.42, *P* = 0.001) (Fig. 5B), but the variation between the flower-camouflaged and the yellow-flowered populations was clinal. Specifically, the yellow-flowered population TBS had a distinct volatile composition, whereas that of the flower-camouflaged population PJ overlapped with two yellow-flowered populations HS and HLH.

## DISCUSSION

Our results confirmed that *F. delavayi* relies on pollinators for seed production. Reproductive fitness, in terms of fruit set and seed set, in the flower-camouflaged population is similar to that of most yellow-flowered populations. Bumblebees are important pollinators in all four yellow-flowered populations we studied, whereas flies (mainly Anthomyiidae) are the exclusive pollinators of the flower-camouflaged population. The flowers of the camouflaged population were smaller than those of the yellow-flowered populations, suitable for smaller pollinating insects such as Anthomyiidae flies. Camouflaged flowers are indeed visually inconspicuous to bumblebee and fly pollinators, but olfactory cues may be used by flies to locate such flowers. The higher visitation frequency of flies ensured seed production in the flower-camouflaged population. Collectively, our results revealed that a pollinator shift from bumblebees to flies compensates for the reproductive success of *F. delavayi* with camouflaged flowers.

### Geographical floral trait variation and pollinator shift

Geographical floral trait variation is common in nature (Harder and Barrett, 2006), but is not necessarily accompanied by a pollinator shift. For example, the floral colour pattern of *Gorteria diffusa* varies among populations, but all populations were primarily pollinated by a single species of bee fly (Ellis and Johnson, 2009). For *Anagallis arvensis* with floral colour dimorphism, morph frequency is associated with climatic factors rather than pollinator assemblage (Arista *et al.*, 2013). However, a pollinator shift does occur in some cases. Floral scent and spur length of the orchid *Eulophia parviflora* differ between populations adapted to bee and beetle pollination, respectively (Peter and Johnson, 2014). Floral colour change controlled by a single locus could induce pollinator shifts between bees and hummingbirds (Bradshaw and Schemske, 2003). Our results showed a pollinator shift associated with floral trait divergence. In all four yellow-flowered populations, bumblebees are important pollinators. Indeed for population TBS, bumblebees are the only pollinators observed. In contrast, in the flower-camouflaged population PJ, only flies were observed as pollinators over three successive years.

Although bumblebees are well-known pollinators in the alpine zone (Bingham and Orthner, 1998), flies also play important roles in such cold environments. Anthomyiid insects, the main pollinators found in the flower-camouflaged population, have also been reported as the pollinators of other alpine and arctic flora (Kevan, 1972; Wagner *et al.*, 2016). The pollination efficiency of flies was considered to be relatively low, as flies are smaller than bumblebees and remain longer within the same flower rather than visiting different individuals. However, their higher visitation frequency often results in high reproductive fitness (reviewed in Inouye *et al*., 2015). Our observations confirmed this finding. Flies visited the camouflaged flowers at a much higher frequency than bumblebees in other populations, resulting in a high fruit set and seed set.

### Sensory ecology and the evolution of camouflaged flowers

Bumblebees have trichromatic colour vision with maximum sensitivity at 328, 428 and 536 nm (Peitsch *et al.*, 1992), covering the ultraviolet, blue and green regions. Based on estimation of colour perception, the yellow flowers of *F. delavayi* are conspicuous to bumblebees, whereas the camouflaged colours of our studied species are difficult for them to distinguish from the rock background. This may explain why bumblebees never visit the flower-camouflaged *F. delavayi*, although they were common and visited many other co-flowering plants in the same location. It has been shown that flies (hoverflies) are capable of discerning small colour differences (Hannah *et al*., 2019), but visual signals only play limited roles in foraging of many Diptera taxa (Roy and Raguso, 1997). Many fly-pollinated flowers are dull coloured, such as yellow-green, green, brown, or even almost black (Shrestha *et al*., 2016). Scent is a more crucial cue for them to locate these visually inconspicuous flowers (Johnson, 1995; Johnson *et al*., 2001; Shuttleworth and Johnson, 2012; Heiduk *et al*., 2023). A noticeable example is *Fritillaria camtschatcensis* (with dark flowers, commonly known as the black lily), a congener of *F. delavayi*, which produces unpleasant floral odour and is observed to be pollinated by flies (Li *et al.*, 2023).

Despite sharing a similar flower colour with the black lily (*F. camtschatcensis*), *F. delavayi* has a pleasant floral scent that is distinct from that of the black lily. Our analyses confirmed that *F. delavayi* is a typical bee-pollinated flower, containing abundant terpenoids such as linalool, limonene, α-cubebene and caryophyllene, among others. Previous studies have identified specific compounds, such as linalool, as innate attractants that elicited strong responses in bumblebee antennae (Kubo and Ono, 2014). Another compound, dimethyl phthalate, has been shown to trigger significant electrophysiological responses in bees (Mayer, 1997; Zhang *et al.*, 2022), indicating its potential as an attractant. Furthermore, we noted that some of the odours have a fruity scent (1-undecanol, linalool and β-cubebene), which may function to attract fly pollinators. We also noted that, although there seems to be a clinal variation in floral scent among populations, there is a distinct composition of compounds between the two extremes, namely the only bumblebee-pollinated population TBS and the only fly-pollinated population PJ. Specifically, PJ displays distinctive floral scent compounds, including linalool, α-copaene and *cis*-farnesol, among others, which may contribute to attracting fly pollinators [linalool (Niogret and Epsky, 2018), α-copaene (Robacker *et al.*, 1992), caryophyllene (Jaleel *et al.*, 2019) and *trans*-β-farnesene (Tesh *et al.*, 1992)]. Additionally, there is evidence indicating that the specific combination of linalool, caryophyllene, α-farnesene (these three compounds were uniquely found in the PJ population) and humulene has the potential to attract the cabbage fly, a member of the Anthomyiidae (Kergunteuil *et al.*, 2015). It is possible that the camouflaged flowers produce certain compounds that repel bumblebees, but we did not find such components in the PJ population.

Although camouflaged leaves are found in several populations of *F. delavayi* (Niu *et al*., 2021), camouflaged flowers were only found in the PJ population in the present study. This may imply ongoing floral trait evolution. Initially, camouflage may have evolved under the pressure of higher survivorship (Niu *et al*., 2014). Individuals with both cryptic leaves and cryptic flowers provide better camouflage, but inevitably decrease their visual attraction for their primary pollinators, bumblebees. Under such circumstances, pollinators may shift from more visual-dependent bumblebees to more scent-dependent flies. Although flies are less efficient pollinators than bumblebees, they visited the camouflaged flowers more frequently, which eventually guaranteed reproductive success.

One possibility that we have not examined here is the thermal effect of floral colour. Cryptic flowers are darker than yellow flowers, which might lead to higher floral temperatures in certain conditions and enhance reproductive success (Sapir *et al.*, 2006; Lacey *et al.*, 2010; but see Gao *et al.*, 2019). This aspect warrants further focused investigation.

## SUPPLEMENTARY DATA

Supplementary data are available at *Annals of Botany* online and consist of the following.

Fig. S1: Reproductive success of *F. dedavayi* across populations in 2022, in terms of fruit set (A, with sample sizes shown within bars), seed set (B) and seed number per plant (C). Fig. S2: The size of *Fritillaria delavayi* flowers (in terms of pistil and stamen length) and pollinators (in terms of body length). Table S1: Information on the studied populations of *Fritillaria delavayi*. Table S2: Floral morphological characteristics (mean ± s.e.) in five populations of *Fritillaria delavayi*, analysed by one-way ANOVA. Table S3: Mean relative amounts (%) of floral scent volatiles of *F. delavayi* from five populations (mean ± s.e.).

mcae075_suppl_Supplementary_Data

## Data Availability

Data are available in ScienceDB at: 10.57760/sciencedb.15515
