# Supplementary material for: Pollinator shift ensures reproductive success in a camouflaged alpine plant
Source: Ann Bot. 2024 May 9;134(2):325–36. doi: 10.1093/aob/mcae075 (PMC11232517; doi:10.1093/aob/mcae075)
Supplement: mcae075_suppl_Supplementary_Data [file mcae075_suppl_supplementary_data.docx]

**Supplementary data**


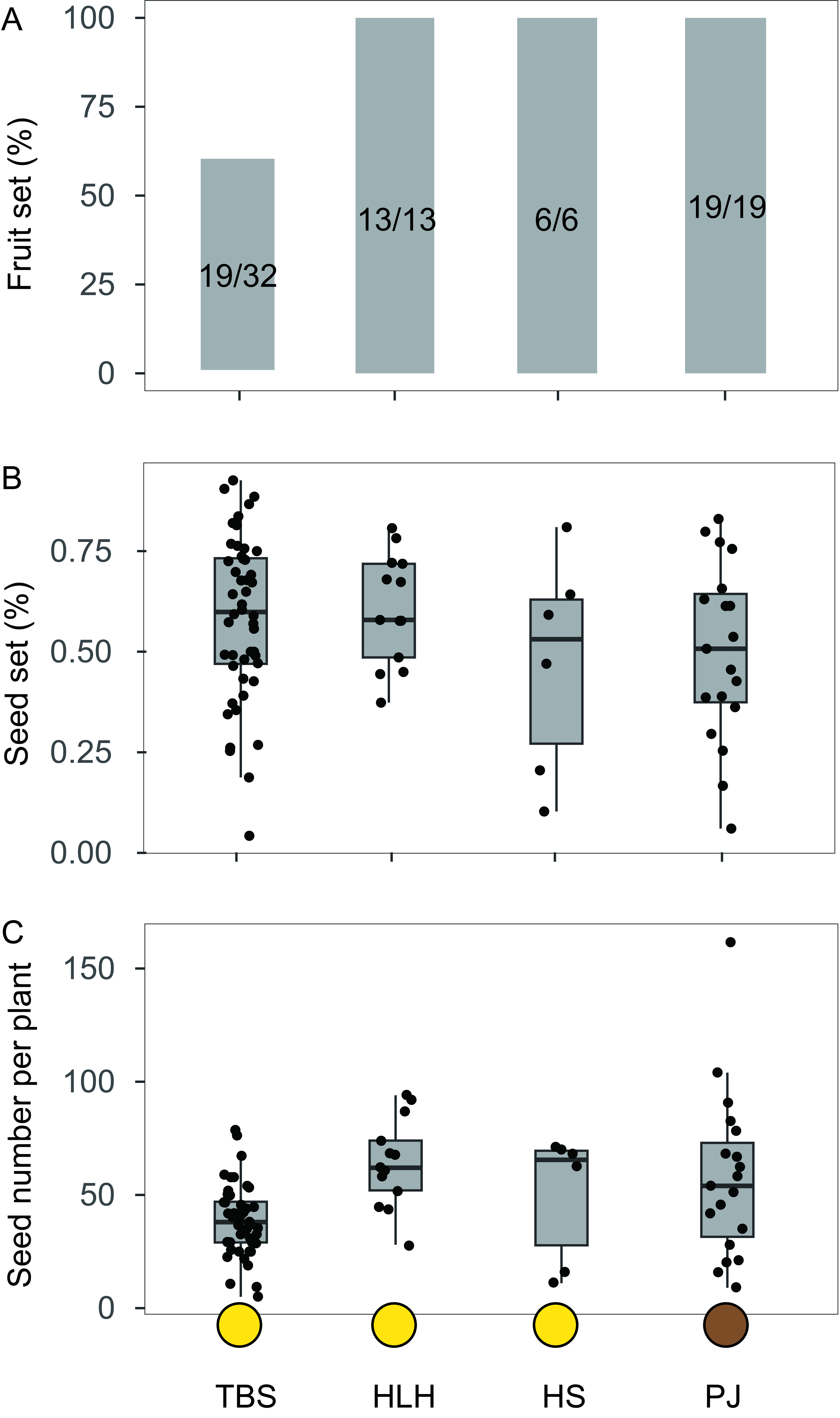


**Figure S1.** Reproductive success of *F. dedavayi* across populations in the year of 2022, in terms of fruit set (A, with sample sizes shown in bars), seed set (B) and seed number per plant (C). Yellow circles represent non-camouflaged populations. Bars indicate standard errors. Reproductive fitness in the flower-camouflaged population PJ is not significantly lower than any of the yellow-flowered population (TBS, HLH and HS).





**Figure S2.** The size of *Fritillaria delavayi* flowers (in terms of pistil and stamen length) and pollinators (in terms of body length). Yellow and brown circles at the bottom represent non-camouflaged and flower-camouflaged populations, respectively. Numbers in brackets are sample sizes. Bars indicate standard errors. Note that pistil and stamen in PJ population are shorter, and Anthomyiidae flies, the only pollinators in flower-camouflaged population PJ, are much smaller than bumblebees.

**Table S1.** Information on the studied populations of *Fritillaria delavayi*.

| **Population** | **Abbreviation** | **Locality** | **Longitude (E)** | **Latitude (N)** | **Elevation (m)** | **Floral colour** |
| --- | --- | --- | --- | --- | --- | --- |
| Tianbaoshan | TBS | NW Yunnan province, China | 99.89 | 27.60 | 3800 | yellow-green |
| Huluhai | HLH | NW Yunnan province, China | 99.95 | 28.53 | 4400 | yellow |
| Hongshan | HS | NW Yunnan province, China | 99.92 | 28.16 | 4200 | yellow |
| Puyong | PY | NW Yunnan province, China | 99.93 | 28.41 | 4600 | yellow |
| Pujing | PJ | NW Yunnan province, China | 99.01 | 28.48 | 4800 | brown |

**Table S2.** The floral morphological characteristics (mean ± s.e.) in five populations of *Fritillaria delavayi*, analysed by one-way ANOVA.

| **Floral characters** | **TBS** | **HLH** | **HS** | **PY** | **PJ** | **F** | **df** | **P** |
| --- | --- | --- | --- | --- | --- | --- | --- | --- |
| Flower diameter | 21.99±0.48^c^ | 25.10±0.88^ab^ | 27.43±0.88^a^ | 24.26±0.74^bc^ | 15.51±0.40^d^ | 39.7 | 4 | < 0.001 |
| Perianth length | 34.90±0.75^c^ | 43.71±0.87^a^ | 43.00±1.01^a^ | 39.21±1.03^b^ | 24.37±0.48^d^ | 69.14 | 4 | < 0.001 |
| Perianth width | 14.04±0.23^c^ | 18.71±0.60^a^ | 19.97±0.68^a^ | 15.92±0.40^b^ | 11.95±0.22^d^ | 65.94 | 4 | < 0.001 |
| Pistil length | 25.83±0.49^d^ | 30.03±0.63^a^ | 29.89±0.75^ac^ | 27.54±0.53^cd^ | 14.33±0.45^b^ | 110.4 | 4 | < 0.001 |
| Stamen length | 20.89±0.38^c^ | 25.81±0.80^a^ | 24.18±0.62^ab^ | 22.69±0.65^bc^ | 11.20±0.29^d^ | 94.22 | 4 | < 0.001 |
| Stamen-pistil distance | 5.09±0.53^a^ | 4.22±0.50^a^ | 5.71±0.75^a^ | 4.85±0.38^a^ | 3.27±0.25^a^ | 2.165 | 4 | = 0.082 |
| Sample size | 24 | 11 | 11 | 16 | 13 |  |  |  |

Different letters indicate significant difference between populations (*p*-value should be less than 0.05 at a minimum, Tukey's tests).

**Table S3.** Mean relative amounts (%) of floral scent volatiles of *F. delavayi* from five populations (Mean ± S.E.).

| **Compounds** | **Populations** | | | | | **Odor description** | **References** |
| --- | --- | --- | --- | --- | --- | --- | --- |
|  | ***HS*** | ***TBS*** | ***PY*** | ***HLH*** | ***PJ*** |  |  |
| Samples of floral scents from different individuals | (*n* = 5) | (*n* = 5) | (*n* = 5) | (*n* = 4) | (*n* = 5) |  |  |
| Number of compounds | 10 | 10 | 7 | 6 | 21 |  |  |
| ***Fatty acid derivatives*** |  |  |  |  |  |  |  |
| Pristane | 14.84±5.56 | 6.22±3.46 | 37.45±6.75 | 20.14±8.64 | 3.11±1.76 | Sweet | (Mario Turchini *et al.*, 2004) |
| Octadecane | 6.05±2.59 | — | — | 2.98±2.58 | — | Clean fragrance | (Li *et al.*, 2022) |
| n-hexadecanoic acid | 17.90±5.22 | — | — | — | — | Sweet | (Zamanhuri *et al.*, 2021) |
| (Z)-4-Tetradecene | 1.50±1.34 | — | — | — | — | No data | — |
| Undecane, 3,5-dimethyl- | — | 2.57±2.3 |  | — | — | No data | — |
| 2,6,10-trimethyl-Pentadecane | — | — | 4.45±3.98 | — | — | Sweet | (Tanchotikul and HSIEH, 1989) |
| 1-Undecanol | — | — | — | 4.53±3.92 | — | Floral, sweet, fruity | (He *et al.*, 2020, Welke *et al.*, 2022) |
| ***Benzenoids*** |  |  |  |  |  |  |  |
| Styrene | 7.56±4.15 | 26.97±10.48 | — | — | 7.22±6.46 | Sweet balsam floral | (Yamaguchi and Shibamoto, 1980) |
| Diisobutyl phthalate | 26.29±8.30 | — | 2.75±2.46 | 24.88±8.30 | 13.09±2.23 | Slight aromatic odor | (Zhu *et al.*, 2015) |
| Benzene, 1,3-dimethyl- | 1.72±1.54 | 5.42±4.84 | — | — | 6.23±5.57 | Aromatic odor | (Wang *et al.*, 2021) |
| Dimethyl phthalate | — | 16.77±6.20 | 23.97±6.60 | 20.25±7.40 | 1.01±0.90 | Aromatic odor | (Yang *et al.*, 2022) |
| Ethylbenzene | — | 2.63±2.35 | — | — | 4.10±3.67 | Sweet | (Wang *et al.*, 2020) |
| p-Xylene | — | 3.44±3.07 | — | — | — | Aromatic odor | (Feng *et al.*, 2021) |
| ***Nitrogen-containing compounds*** |  |  |  |  |  |  |  |
| 3-Pyridinecarboxaldehyde | 10.50±9.39 | — | — | 19.23±16.66 | — | No data | — |
| 4-Pyridinecarboxaldehyde | — | — | 19.87±11.70 | — | — | No data | — |
| ***Terpenoids*** |  |  |  |  |  |  |  |
| Linalool | 10.9±6.52 | — | 5.67±3.20 | — | 1.42±0.90 | Floral, woody, fruity | (Zhang *et al.*, 2019) |
| Limonene | 2.75±2.46 | 2.87±2.57 | — | — | 5.24±4.68 | Lemon-like | (Gupta *et al.*, 2021) |
| α-Cubebene | — | — | — | — | 1.83±1.64 | Spice-like aroma | (Tangpao *et al.*, 2018) |
| β-Cubebene | — | — | — | — | 1.17±0.77 | Citrus, fruity | (Freitas *et al.*, 2020) |
| Caryophyllene | — | — | — | — | 7.42±3.65 | Woody, spicy | (Salvador *et al.*, 2017) |
| D-Germacrene | — | — | — | — | 9.77±6.16 | Woody, spicy | (Acree, 2004) |
| Farnesal | — | — | — | — | 5.76±2.47 | No data | — |
| Sabinene | — | 18.78±9.44 | — | — | 3.11±2.78 | Woody | (Xiao *et al.*, 2016) |
| L-β-Pinene | — | 14.34±7.88 | — | — | — | Woody, piney | (Mehta *et al.*, 2018) |
| 1-Nonene | — | — | 5.85±5.23 | — | — | No data | — |
| ψ-Limonene | — | — | — | — | 0.41±0.36 | Lemon-like | (Wang *et al.*, 2017) |
| α-Copaene | — | — | — | — | 0.56±0.50 | Woody, spicy | (Li *et al.*, 2017) |
| trans-β-Farnesene | — | — | — | — | 0.38±0.34 | Sweet | (Choi and Sawamura, 2000) |
| α-Farnesene | — | — | — | — | 0.46±0.41 | Citrus-like, fruity | (Wang *et al.*, 2024) |
| δ-Cadinene | — | — | — | — | 0.27±0.24 | Woody, thyme, medicine | (Acree, 2004) |
| cis-Farnesol | — | — | — | — | 24.22±10.87 | Sweet | (Miyazawa *et al.*, 2015) |
| α-Terpinolene | — | — | — | — | 3.23±2.89 | Floral, sweet | (Meshkatalsadat *et al.*, 2010) |

**LITERATURE CITED**

**Acree T.** **2004**. Flavornet and human odor space. *http://www. flavornet. org/flavornet. html*.

**Choi H-S, Sawamura M.** **2000**. Composition of the essential oil of *Citrus tamurana* Hort. Ex Tanaka (Hyuganatsu). *Journal of Agricultural and Food Chemistry* **48**: 4868-4873. doi:10.1021/jf000651e.

**Feng M, Dai Z, Yin Z, Wang X, Chen S, Zhang H.** **2021**. The volatile flavor compounds of Shanghai smoked fish as a special delicacy. *Journal of Food Biochemistry* **45**: e13553. doi:10.1111/jfbc.13553.

**Freitas TP, Taver IB, Spricigo PC, do Amaral LB, Purgatto E, Jacomino AP.** **2020**. Volatile compounds and physicochemical quality of four jabuticabas (*Plinia* sp.). *Molecules* **25**: 4543. doi:10.3390/molecules25194543.

**Gupta A, Jeyakumar E, Lawrence R.** **2021**. Journey of limonene as an antimicrobial agent. *Journal of Pure & Applied Microbiology* **15**: 1094-1110. doi:10.22207/JPAM.15.3.01.

**He Y, Liu Z, Qian M, Yu X, Xu Y, Chen S.** **2020**. Unraveling the chemosensory characteristics of strong-aroma type Baijiu from different regions using comprehensive two-dimensional gas chromatography–time-of-flight mass spectrometry and descriptive sensory analysis. *Food Chemistry* **331**: 127335. doi:10.1016/j.foodchem.2020.127335.

**Li Y, Ran W, He C*, et al.*** **2022**. Effects of different tea tree varieties on the color, aroma, and taste of Chinese Enshi green tea. *Food Chemistry: X* **14**: 100289. doi:10.1016/j.fochx.2022.100289.

**Li Y, Wan Y, Sun Z*, et al.*** **2017**. Floral scent chemistry of *Luculia yunnanensis* (Rubiaceae), a species endemic to China with sweetly fragrant flowers. *Molecules* **22**: 879. doi:10.3390/molecules22060879.

**Mario Turchini G, Giani I, Caprino F, Maria Moretti V, Valfrè F.** **2004**. Discrimination of origin of farmed trout by means of biometrical parameters, fillet composition and flavor volatile compounds. *Italian Journal of Animal Science* **3**: 123-140. doi:10.4081/ijas.2004.123.

**Mehta PK, de Sousa Galvão M, Soares AC, Nogueira JP, Narain N.** **2018**. Volatile constituents of Jambolan (*Syzygium cumini* L.) fruits at three maturation stages and optimization of HS-SPME GC-MS method using a central composite design. *Food Analytical Methods* **11**: 733-749. doi:10.1007/s12161-017-1038-4.

**Meshkatalsadat MH, Safaei-Ghomi J, Moharramipour S, Nasseri M.** **2010**. Chemical characterization of volatile components of *Tagetes minuta* L. cultivated in south west of Iran by nano scale injection. *Digest Journal of Nanomaterials and Biostructures* **5**: 101-106.

**Miyazawa M, Nakashima Y, Nakahashi H*, et al.*** **2015**. Volatile compounds with characteristic odor of essential oil from *Magnolia obovata* leaves by hydrodistillation and solvent-assisted flavor evaporation. *Journal of Oleo Science* **64**: 999-1007. doi:10.5650/jos.ess15114.

**Salvador ÂC, Silvestre AJ, Rocha SM.** **2017**. Unveiling elderflowers (*Sambucus nigra* L.) volatile terpenic and norisoprenoids profile: effects of different postharvest conditions. *Food Chemistry* **229**: 276-285. doi:10.1016/j.foodchem.2017.02.037.

**Tanchotikul U, HSIEH TCY.** **1989**. Volatile flavor components in crayfish waste. *Journal of Food Science* **54**: 1515-1520. doi:10.1111/j.1365-2621.1989.tb05149.x.

**Tangpao T, Chung H-H, Sommano SR.** **2018**. Aromatic profiles of essential oils from five commonly used Thai basils. *Foods* **7**: 175. doi:10.3390/foods7110175.

**Wang C, Zhang C, Kong Y*, et al.*** **2017**. A comparative study of volatile components in Dianhong teas from fresh leaves of four tea cultivars by using chromatography-mass spectrometry, multivariate data analysis, and descriptive sensory analysis. *Food Research International* **100**: 267-275. doi:10.1016/j.foodres.2017.07.013.

**Wang Q, Shen J, Zeng B, Wang H.** **2021**. Research on VOCs and odor from heartwood and sapwood of paper mulberry (*Broussonetia papyrifera* (L.) Vent.) with different moisture content. *Wood Science and Technology* **55**: 1153-1170. doi:10.1007/s00226-021-01292-8.

**Wang Q, Zeng B, Shen J, Wang H.** **2020**. Effect of lacquer decoration on VOCs and odor release from *P. neurantha* (Hemsl.) Gamble. *Scientific Reports* **10**: 9565. doi:10.1038/s41598-020-66724-0.

**Wang X, Cao J, Cheng X*, et al.*** **2024**. UV-B application during the aeration process improves the aroma characteristics of oolong tea. *Food Chemistry* **435**: 137585. doi:10.1016/j.foodchem.2023.137585.

**Welke JE, Nicolli KP, Hernandes KC, Biasoto ACT, Zini CA.** **2022**. Adaptation of an olfactometric system in a GC-FID in combination with GCxGC/MS to evaluate odor-active compounds of wine. *Food Chemistry* **370**: 131004. doi:10.1016/j.foodchem.2021.131004.

**Xiao Z, Ma S, Niu Y, Chen F, Yu D.** **2016**. Characterization of odour‐active compounds of sweet orange essential oils of different regions by gas chromatography‐mass spectrometry, gas chromatography‐olfactometry and their correlation with sensory attributes. *Flavour and Fragrance Journal* **31**: 41-50. doi:10.1002/ffj.3268.

**Yamaguchi K, Shibamoto T.** **1980**. Volatile constituents of the chestnut flower. *Journal of Agricultural and Food Chemistry* **28**: 82-84. doi:10.1021/jf60227a021.

**Yang H, An W, Wang F*, et al.*** **2022**. Integrated transcriptomic, metabolomic, and physiological analyses reveal new insights into fragrance formation in the heartwood of *Phoebe hui*. *International Journal of Molecular Sciences* **23**: 14044. doi:10.3390/ijms232214044.

**Zamanhuri NA, Abd Rahman N, Bakar NFA.** **2021**. Effect of microwave power and extraction time on crude palm oil quality using microwave-assisted extraction process. *International Journal of Renewable Energy Development* **10**: 495-505. doi:10.14710/ijred.2021.35402.

**Zhang WJ, Liu C, Yang RJ*, et al.*** **2019**. Comparison of volatile profiles and bioactive components of sun-dried Pu-erh tea leaves from ancient tea plants on Bulang Mountain measured by GC-MS and HPLC. *Journal of Zhejiang University. Science. B* **20**: 563-575. doi:10.1631/jzus.B1800183.

**Zhu Y, Yang T, Shi J*, et al.*** **2015**. Analysis of aroma components in Xihu Longjing tea by comprehensive two-dimensional gas chromatography-time-of-flight mass spectrometry. *Scientia Agricultura Sinica* **48**: 4120-4146. doi:10.3864/j.issn.0578-1752.2015.20.013.
